# Supplementary material for: Content-rich biological network constructed by mining PubMed abstracts
Source: BMC Bioinformatics. 2004 Oct 8;5:147. doi: 10.1186/1471-2105-5-147 (PMC528731; doi:10.1186/1471-2105-5-147)
Supplement: Additional File 5 — The original Chilibot query results of the term "long-term potentiation (LTP)" and 22 other terms, limiting the latest references analyzed to the years 1990, 1995, 2000, and 2004. [file 1471-2105-5-147-S5.bz2 › chilibotAdditionalFile5/ltp2000/html/TAU_ACTIN.html]

 


 **TAU** and **ACTIN** 
  
Found 130 abstracts in PubMed,  **30 abstracts were retrieved and analyzed**.  


---

 Search Google  |
 PDF files only 
|  EDU domain only 

---

**Interactive relationship** (e.g. stimulation, inhibition, etc)

- Subpopulations of  **tau**  interact with microtubules and  **actin**  filaments in various cell types.  Ref: 10232926 Cell Biochem Funct, 1995
- **Actin**  disruption alters the localization of  **tau**  in the growth cones of cerebellar granule neurons.  Ref: 10893194 J Cell Sci, 2000
- There is evidence that  **actin**  microfilaments F  **actin**  are involved in the elaboration of  **tau**  mediated process outgrowth in Sf9 cells.  Ref: 10223633 Cell Motil Cytoskeleton, 1999
- The heterogeneous family of  **tau**  proteins interacts with microtubules,  **actin**  filaments, and intermediate filaments.  Ref: 10842324 J Cell Biochem, 2000
- These results suggest the possible in vivo roles of subsets of  **tau**  protein in modulating the interactions between microtubules and  **actin**  filaments.  Ref: 10232926 Cell Biochem Funct, 1995
- Immunoprecipitation studies along with immunolocalization experiments in cell lines containing  **tau**  like components supported the findings on the roles of  **tau**  isospecies as linkers between tubulin in the microtubular structure with  **actin**  filaments.  Ref: 10232926 Cell Biochem Funct, 1995
- Depletion of  **tau**  also was not accompanied by a reorganization of other major MT associated proteins or  **actin**  filaments in these neurons.  Ref: 9786973 J Neurosci, 1998
- Interestingly, in certain types of cells, antibody reactive  **tau**  isospecies were detected by immunofluorescence with a discrete distribution pattern along  **actin**  filaments, which was affected by cytochalasin disruption of the  **actin**  filament network.  Ref: 10232926 Cell Biochem Funct, 1995
- However,  **tau**  0 was significantly decreased upon treatment of fibroblasts with inhibitors of  **actin**  polymerization or myosin function.  Ref: 10192936 Eur Biophys J, 1999

**Parallel relationship** (e.g. studied together, co-existance, homology, etc.)

- The neuronal proteins  **Tau**  and MAP2 use homologous C terminal MT binding regions MTBRs to interact with microtubules, F  **actin** , and intermediate filaments.  Ref: 10527895 Mol Cell Biol Res Commun, 1999
- In the present study, we determined the location of the dephosphorylated form of the microtubule associated protein  **tau**  dtau within the growth cones of newly forming axons and examined whether this localization was influenced by the  **actin**  cytoskeleton.  Ref: 10893194 J Cell Sci, 2000
- Other cytoskeletal proteins, including  **actin** , microtubule associated protein, and  **tau** , are not influenced by chronic ECS administration.  Ref: 10611639 Synapse, 2000
- In cultures exposed to mild glutamate toxicity,  **tau**  mRNA expression, not beta  **actin** , is enhanced in stressed neurons.  Ref: 10461544 Drug Metab Rev, 1999
- Since cytoskeletal proteins play crucial roles in promoting neurite outgrowth, we examined the protein levels of some of these proteins that are associated with neurite outgrowth beta  **actin** , gamma  **actin** , alpha tubulin, MAP2 and  **tau** .  Ref: 11020545 Brain Res Dev Brain Res, 2000
- This suggests that MAP2b exerts an effect on F  **actin**  stability at an earlier stage of infection than  **tau** .  Ref: 10223633 Cell Motil Cytoskeleton, 1999
- When the remaining cytoskeletal pellet was treated with cytochalasin D to allow depolymerization of  **actin**  filaments, only  **tau**  isoforms were extracted.  Ref: 10232926 Cell Biochem Funct, 1995
- A mathematical description of the dynamics of the  **actin**  cytoskeleton predicts that the evolution of fluorescence in PAF and FRAP experiments depends simultaneously on the diffusion coefficient of  **actin**  monomer, D, the fraction of  **actin**  in filaments, FF, and the lifetime of  **actin**  filaments,  **tau** , Biophys.  Ref: 9746549 Biophys J, 1998
- Collectively, these results indicate that MAP2b could impair the ability of MAP2c and  **tau**  to redistribute F  **actin**  in Sf9 cells, thereby decreasing their capacity to induce process formation.  Ref: 10223633 Cell Motil Cytoskeleton, 1999
- We compared the effects of MAP2b and MAP2c with the effects of  **tau**  on F  **actin**  distribution and stability in Sf9 cells.  Ref: 10223633 Cell Motil Cytoskeleton, 1999
